# Sustained Loss of *Bdnf* Affects Peripheral but Not Central Vestibular Targets

**DOI:** 10.3389/fneur.2021.768456

**Published:** 2021-12-16

**Authors:** Karen L. Elliott, Jennifer Kersigo, Jeong Han Lee, Ebenezer N. Yamoah, Bernd Fritzsch

**Affiliations:** ^1^Department of Biology, University of Iowa, Iowa City, IA, United States; ^2^Department of Physiology, School of Medicine, University of Nevada, Reno, NV, United States; ^3^Department of Otolaryngology, University of Iowa, Iowa City, IA, United States

**Keywords:** *Bdnf*, *Pax2-Cre*, hair cells, lateral vestibular neurons, nodule, uvula, fastigial neurons, vestibular ganglion neurons

## Abstract

The vestibular system is vital for proper balance perception, and its dysfunction contributes significantly to fall-related injuries, especially in the elderly. Vestibular ganglion neurons innervate vestibular hair cells at the periphery and vestibular nuclei and the uvula and nodule of the cerebellum centrally. During aging, these vestibular ganglion neurons degenerate, impairing vestibular function. A complete understanding of the molecular mechanisms involved in neurosensory cell survival in the vestibular system is unknown. Brain-derived neurotrophic factor (BDNF) is specifically required for the survival of vestibular ganglion neurons, as its loss leads to early neuronal death. *Bdnf* null mice die within 3 weeks of birth, preventing the study of the long-term effects on target cells. We use *Pax2*-cre to conditionally knock out *Bdnf*, allowing mice survival to approximately 6 months of age. We show that a long-term loss of *Bdnf* leads to a significant reduction in the number of vestibular ganglion neurons and a reduction in the number of vestibular hair cells. There was no significant decrease in the central targets lateral vestibular nucleus (LVN) or the cerebellum at 6 months. This suggests that the connectivity between central target cells and other neurons suffices to prevent their loss despite vestibular hair cell and ganglion neuron loss. Whether the central neurons would undergo eventual degeneration in the absence of *Bdnf* remains to be determined.

## Introduction

The vestibular system is paramount in the perception of balance, and its dysfunction is a significant contributor to fall-related injuries, especially amongst the elderly (1–4). Behavioral assessment of vestibular function confirms an age-dependent decline (5–7). Age-related vestibular defects correlate well with the loss of vestibular hair cells (8) as well as loss of vestibular ganglion neurons (9). In addition, there is a reduction of vestibular nucleus neurons, particularly the lateral vestibular neurons (1), which are a significant output to the spinal cord (10–13). The peripheral vestibular system is housed in the dorsal portion of the inner ear. It comprises five sensory epithelia in mammals: the utricle and saccule for linear acceleration perception and the anterior, horizontal, and posterior semicircular canal cristae for angular acceleration perception. Vestibular ganglion neurons innervate mechanosensory hair cells located within each sensory epithelia (14, 15), sending central processes to the vestibular nuclei in the hindbrain and the cerebellum (16). Centrally, higher-order integration occurs to maintain posture and gaze (17). In the hindbrain, the lateral vestibular nuclei (LVN), medial vestibular nuclei (MVN), superior vestibular nuclei (SVN), and spinal vestibular nuclei (SpVN) are innervated by vestibular ganglion neurons that have different terminal zones at the periphery (18, 19). The LVN neurons, which are derived from rhombomere 4 of the hindbrain, innervate ipsilateral spinal motor nuclei exclusively (11, 12). These neurons are involved in the early activation of extensor muscles and initiate co-activation of extensors and flexors during balancing (20). Within the cerebellum, vestibular ganglion neurons project to the nodule and uvula. An intricate anatomic connection between the nodule and uvula of the cerebellum has been shown (16, 21–24). The unipolar brush cells [UBC; (18, 19)] are highly present in the nodule, uvula, and flocculus (25, 26), providing unique input feedback and feedforward interactions with the vestibular ganglion neurons (16, 21, 27, 28). Additionally, the fastigial nucleus neurons project to the uvula and nodule (18, 29, 30) as well as to the LVN, based on tracing studies (30–32), providing another layer of feedback and interconnection. Data in mice show an age-related decline of vestibular ganglion neurons (33), in line with the reduced vestibular functionality in humans (34) and subsequent fall-related injuries (35, 36). Compounding neuronal loss alone is that many target cells depend upon sustained innervation for their maintenance (37, 38). Still, a complete understanding of the molecular mechanisms involved in the vestibular system neurosensory cell survival is not entirely known.

Neurons of the inner ear require neurotrophins for survival. Neurotrophins signal through Trk protein-tyrosine kinase receptors (39). In the inner ear, *brain-derived neurotrophic factor* (*Bdnf*) and *neurotrophic factor 3* (*Ntf3*), which signal through the *Ntrk2* (*TrkB*) and *Ntrk3* (*TrkC*) receptors, respectively, are expressed, and in the absence of both neurotrophins (38) or receptors (40), no neurons survive. Vestibular ganglion neurons, which express *Ntrk2* require *Bdnf* expressed by vestibular hair cells (41–44). In addition, the Ntrk2 receptor is essential for vestibular ganglion neuron survival (45, 46). Complete loss of *Bdnf* resulted in a reduction in innervation to the sensory epithelia as early as embryonic day (E) 13.5, and by postnatal day (P) 15, very few vestibular ganglion neurons remained (41). However, loss of *Bdnf* did not affect the hair cell and central neuron targets of the vestibular ganglion neurons at these stages (41). Given the early lethality of *Bdnf* null mice [P21 (41)], any long-term effect on the loss of *Bdnf* on vestibular ganglion neuron peripheral or central target cells has not been assessed. In the present study, we use *Pax2-cre* to conditionally knockout *Bdnf* in the ear (47), allowing us to reliably raise most mice to at least 6 months of age. Here we ask how a sustained loss of *Bdnf* affects the peripheral targets (vestibular hair cells) and the central targets (vestibular nuclei and cerebellum). Our results show that a long-term loss of *Bdnf* leads to a significant reduction in the number of vestibular ganglion neurons and a reduction in the number of vestibular hair cells. However, even with a 6-month loss of *Bdnf*, there is no significant reduction in the central targets of the LVN or the cerebellum.

## Materials and Methods

### Mouse Models and Genotyping

To generate the conditional *Bdnf* knockout mice (CKO) we crossed the *Pax2*-cre line (38, 47) with the *Bdnf* floxed line (48); Jackson Laboratory Stock 021055). Here *Bdnf* ^*f*/*f*^ *Pax2*-cre {or *Bdnf* ^*f*/*f*^ *Ntf3* ^*f*/+^ *Pax2*-cre when noted [*Ntf3* floxed line; (49)]} were used as the conditional knockout mutants, and littermate *Bdnf* ^f/+^ *Pax2*-cre and *Bdnf* ^+/+^ were used as wild-type controls. Both male and female mice were used. Genotyping was confirmed by PCR analysis on tail DNA with *Bdnf*- and *Ntf3*-specific primers (BDNF loxp 3651 For 5'-GTG TGT TCA CTT GCT TAG AAA CCG-3' and BDNF loxp 4125 Rev: 5'-CCA GTT ATG TCG TCG TCA GAC CTC TC-3'; NT3 5644 loxp For 5'-GCA AGC AAT CAG AAG ACC AGT GC−3' and NT3 8135 loxp Rev: 5'-TCA TTG GCT GGA ACT CTT ACC TGG-3') and general *Cre* primers (Cre1: 5'-CCT GTT TTG CAC GTT CAC CG-3'; Cre3: 5'-ATG CTT CTG TCC GTT TGC CG-3'; IMR0042: 5'-CTA GGC CAC AGA ATT GAA AGA TCT-3'; IMR0043: 5'-GTA GGT GGA AAT TCT AGC ATC ATC C-3').

### Fixation and Tissue Preparation

Mice were anesthetized (1.25% tribromoethanol solution; 0.025 ml/g of body weight) and transcardially perfused at various stages with 4% paraformaldehyde in PBS (pH 7.6) with 0.3 M sucrose. The heads were removed, bisected, and stored in 4% PFA at 4°C until further processing. The temporal bones were removed and decalcified in 0.25M EDTA solution (RPI E57020) for up to 5 days with daily solution changes. Decalcified inner ears were washed in PBS and micro-dissected for immunofluorescence. Fixed brain tissue was dissected to isolate the areas of interest (i.e., hindbrain and cerebellum), briefly blotted with paper (Spilfyter, Sigma-Aldrich #Z558591), embedded in 3% agarose (Millipore-Sigma #A0576) using Tissue-Tek disposable Cryomolds (Sakura Finetek #4566 and #4557), and then placed on a flat ice block to set. When hardened, the block/sample was trimmed, mounted, and re-embedded in 3% agarose on the stage of a Compresstome VF-700 microtome (Precisionary Instruments Inc.). The samples were sectioned in the coronal (hindbrain) or parasagittal (cerebellum) plane at 50-μm steps and collected in 0.4% PFA, and temporarily stored at 4°C. Mice were sacrificed at approximately 1 month (P18-P28) and about 6 months (P135-P206) of age.

### Dye Tracing

To investigate the effect of residual loss of vestibular ganglion neurons in control and *Bdnf CKO* mice, different colored lipophilic dye-soaked filter paper wedges (MTTI; NeuroVue Red FS-1002, NeuroVue Maroon FS-1001) were implanted into the utricle/anterior canal cristae/horizontal canal cristae (U, AC, HC) and the saccule (S) of P18-23 *Bdnf* CKO (n=3) and control littermates (n=3) following transcardial perfusion with 4% PFA. In addition, we inserted lipophilic dyes into the uvula and nodule to label the residual innervation of vestibular ganglion neurons in control and *Bdnf* CKO mice (n=3). Dyes diffused for ~19 days at 60°C (16, 50).

### Immunohistochemistry

Whole-mount, dissected vestibular sensory epithelia, and free-floating brain sections were blocked and permeabilized with 5% NHS/0.3% Triton X-100 (Jackson ImmunoResearch 008-000-121) in PBS for 1 h then incubated in primary antibody solution (PBS + 1% NHS + antibodies) for 24–48 h at 4°C. After several PBS washes (3 x 1 hour) at room temperature, the samples were incubated in species-specific secondary antibody solution with DAPI (Sigma-Aldrich D9542, 1 μg/ml) at 4°C for 12–24 h protected from light. Finally, the samples were washed several times in PBS (3 x 1 h) before viewing. Please see Tables 1, 2 for antibody details.

**Table 1:** Primary antibody details.

**Antibody**	**Host**	**Company**	**Product Number**	**Dilution**
anti-Acetylated Tubulin anti-Calbindin	mouse	MilliporeSigma	T7451	1:800
anti-Calbindin	rabbit	Cell Signaling	13176S	1:500
anti-Calretinin	chicken	EnCor Biotechnology	CPCA-Calret	1:1000
anti-Myosin-VIIa	rabbit	Proteus Biosciences	25-6790	1:300
anti-NeuN	rabbit	Cell Signaling	12943	1:500
anti-Neurofilament-H	chicken	MilliporeSigma	AB5539	1:1000
anti-Parvalbumin	guinea pig	Swant	GP72	1:1000

**Table 2:** Secondary antibody details.

**Antibody**	**Company**	**Product Number**	**Dilution**
Goat anti-Chicken IgY (H+L), Alexa Fluor 488	Invitrogen	A-11039	1:500
Goat anti-Chicken IgY (H+L) Alexa Fluor 546	Invitrogen	A-11040	1:500
Goat anti-Guinea Pig IgG (H+L) Alexa Fluor 647	Invitrogen	A-21450	1:500
Goat anti-Mouse IgG (H+L) Alexa Fluor 488	Invitrogen	A-11001	1:500
Goat anti-Mouse IgG (H+L) Alexa Fluor 647	Invitrogen	A-21235	1:500
Goat anti-Rabbit IgG (H+L) Alexa Fluor 488	Invitrogen	A-11008	1:500
Goat anti-Rabbit IgG (H+L) Alexa Fluor 546	Invitrogen	A-11008	1:500

### Imaging

Images were acquired using a Leica SP8 scanning laser confocal microscope, analyzed with Leica LAS X software, and processed with CorelDRAW graphics suite. Images were taken at 1–6 μm thick optical sections to compile a given stack, in up to four different colors (405, 488, 552, and 638 nm laser lines with emission recorded at 430–481 nm, 500–547 nm, 557–625 nm, and 643–776 nm, respectively), and imaged in sequence using three different magnifications (10x with a 0.5 NA; 20x with a 0.95; 63x with a 1.4 NA).

### Quantification

For quantification of vestibular sensory epithelial area, maximum projection confocal TIFF images of the utricle, saccule, and posterior canal crista, labeled with Myo7a antibodies, were exported from Leica LAS X software, with scale bar, and imported into ImageJ software. Each image was calibrated in ImageJ for the number of pixels per 1 μm. The area was calculated using the tracing and measure functions. In addition, the length and width of each utricle, saccule, and posterior canal crista were measured. Four 6-month *Bdnf* CKO and four control littermates were analyzed.

To quantify the number of hair cells within representative areas of the saccule, three 100 μm x 100 μm boxes were drawn within each sensory epithelium using the Leica LAS X software. The number of Myo7a-positive hair cells within each box was counted. The numbers of hair cells within each box were averaged per animal. Four 6-month *Bdnf* CKO and four control littermates were analyzed.

For quantification of the area of the vestibular ganglion, the maximum projection confocal “TIFF images” of lipophilic dye labeling of each ganglion were exported from Leica LAS X software and imported into ImageJ software. Each image was calibrated for the number of pixels per 1 μm, and the area was calculated using the tracing and measure function. In addition, the depth of each ganglion was measured to determine the volume. Percent reduction was calculated by dividing the area and volume measurements from the *Bdnf* CKO mice by the controls. Three P18-P23 *Bdnf* CKO and three control mice were analyzed.

To quantify the number of large neurons in the LVN (Deiter's neurons), images of coronal sections through the LVN (two 50 μm sections per animal), labeled with Calb1, Calb2, and Parvalbumin antibodies or tubulin and neurofilament antibodies, as well as DAPI, were viewed in Leica LAS X software. The diameter of each neuron was determined using Leica LAS X software. The total number of large (>25 μm diameter) LVN neurons per section was manually counted, as these were shown to be preferentially lost with age (33). The number of large LVN neurons was averaged between two sections to give a mean number for each animal. Four 6-month *Bdnf* CKO and three control littermates were analyzed, two sections each.

For quantification of the number of Purkinje cells (Calb1-positive) within the nodule (lobe X) of the cerebellum, 400 μm lines were drawn along the dorsal and ventral portion of the lobe. The central optical section was selected, and the number of Purkinje cells was manually counted along the lines. The average between the number along the two lines was averaged for each animal. To quantify the number of UBCs (Calb2-positive), 50 x 50 μm boxes were drawn in the dorsal and ventral portion of the lobe within the a were manually counted in three individual single optical sections spaced a minimum of 9 μm in depth. The numbers of UBCs within the boxes were averaged for the ventral and dorsal portions to give a mean for each animal. Four 6-month *Bdnf* CKO and four control littermates were analyzed.

The sizes of sampling boxes were selected to maximize areas of interest for each respective tissue quantified but remain within the tissue boundaries. Means and standard errors of the mean were calculated using Microsoft Excel. Statistical significance was performed with two-tailed *t*-tests in Microsoft Excel. Significant differences were determined at *p* < 0.05.

## Results

### Conditional Loss of *Bdnf* Affects Vestibular Hair Cell Survival and Innervation

**Figure 1:**
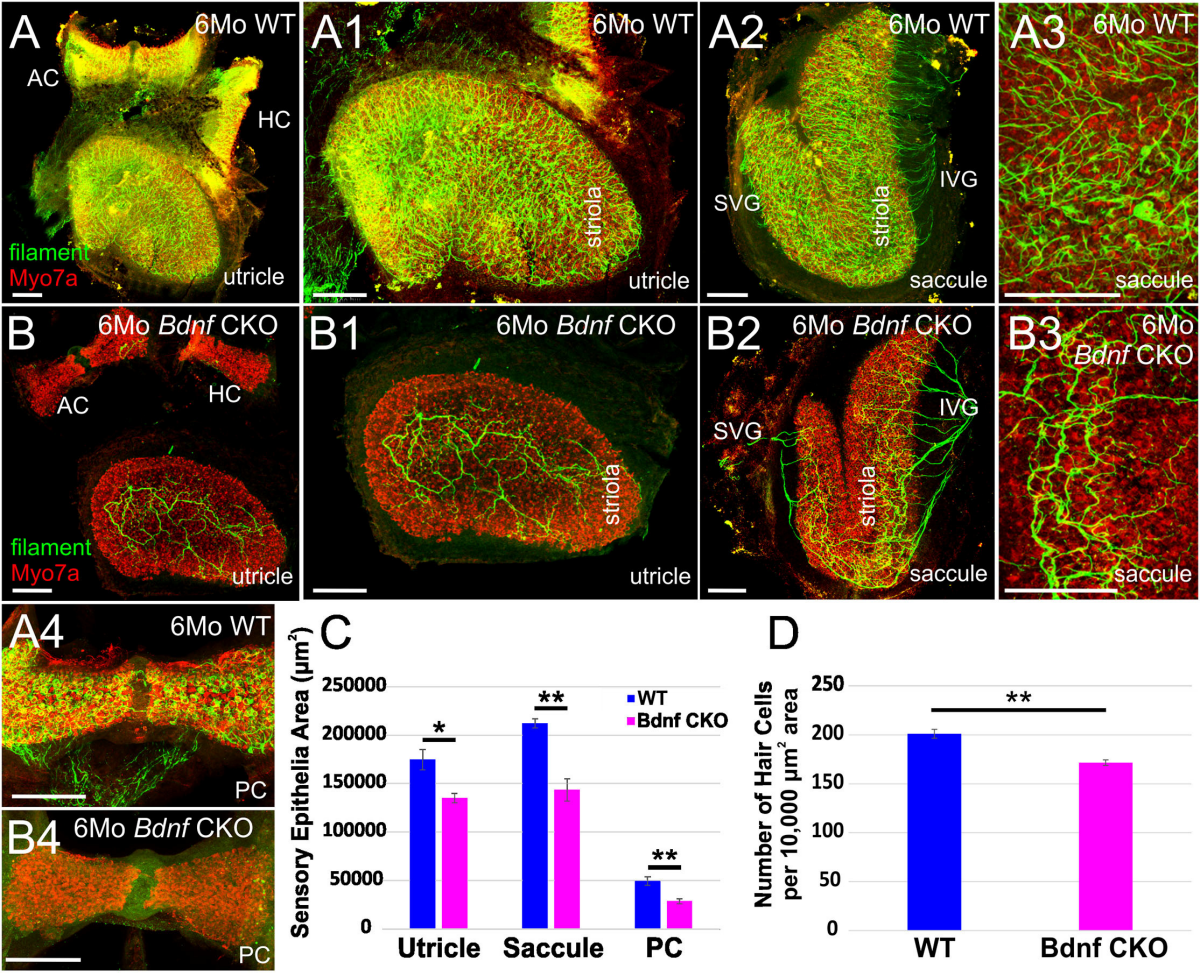
*Bdnf* loss results in a reduction of vestibular hair cells and vestibular ganglion neurons in the inner ear. Overview of the utricles, anterior canal cristae (AC), and horizontal canal cristae (HC) of wild type **(A)** and *Bdnf* CKO **(B)** 6-month-old mice labeled with *Myo7a* (red) and neurofilament (green) antibodies to label hair cells and neurons, respectively. The utricles **(A1,B1)** and saccules **(A2–3, B2–3)** show a clear difference of size and innervation between the wild type **(A1–3)** and *Bdnf* CKO **(B1–3)** mice. The utricle, saccule, and posterior canal crista (PC) of *Bdnf* CKO mice are significantly smaller in area compared with controls **(C)**. Also, the number of hair cells within a 10,000 μm2 area was significantly fewer in *Bdnf* CKO mice than in controls **(D)**. SVG, superior vestibular ganglion; IVG, inferior vestibular ganglion. Bars indicate 100 μm. ^*^p < 0.05; ^**^p < 0.01.

Using antibodies against *Myo7a* to label hair cells and neurofilament to label neurons, we investigated the changes in the vestibular periphery of 6-month-old *Bdnf* CKO mice compared with controls. Unlike control mice in which there is an abundance of innervation to vestibular hair cells in all sensory epithelia, in the *Bdnf* CKO mice, there is a decreased number of peripheral vestibular fibers and terminals to the hair cells within the utricle and saccule (Figures 1A,A3,B,B3) and little to no innervation to the semicircular canal cristae (Figures 1A,A4,B,B4; Supplementary Figures 1A,B2). In particular, the posterior canal crista has virtually no innervation except for the occasional fiber (Figures 1A,B4). In the utricle of the 6-month-old *Bdnf* CKO mice, surviving fibers are mainly concentrated in the center, striolar region with few fibers innervating hair cells at its periphery (Figures 1B–B1). The saccule is unique in its innervation as it is normally innervated by a branch of afferents from the superior vestibular ganglion (SVG) and another branch from the inferior vestibular ganglion (IVG) (Figure 1A2). In the *Bdnf* CKO mice, there is a reduction in the innervation of the saccule from the IVG and few to no direct fibers from the SVG (Figure 1B2; Supplementary Figure 1C). In contrast to the utricle, the remaining fibers innervating the saccule are concentrated at the periphery (Figure 1B2). The two vestibular afferents, type I and type II, terminate on vestibular hair cells either as a calyx or a bouton, respectively. Compared with controls, in *Bdnf* CKO mice, calices were conspicuously absent (Figure 1A3,B3), suggesting a loss of type I vestibular afferents.

Knowing that maintenance of vestibular hair cells requires long-term innervation (38), we examined the sensory epithelia of these 6-month-old *Bdnf* CKO mice using antibodies against *Myo7a* to label the hair cells. Compared with control mice, *Bdnf* CKO mice have smaller sensory epithelia. The total area of the utricle, saccule, and posterior canal crista was calculated. There was a significant reduction in their area in the *Bdnf* CKO mice compared with controls (Figure 1C). While the lengths of the utricle and posterior canal cristae are significantly shorter in the *Bdnf* CKO mice, the length of the saccule is not significantly different from controls (data not shown). However, the widths of all three sensory epithelia are considerably narrower in the *Bdnf* CKO compared with controls (data not shown). We next quantified the density of hair cells in control and *Bdnf* CKO saccules to determine if the reduction in the size of epithelia in the mutants may results from hair cell loss. The mean number of hair cells within a 10,000 μm^2^ area was significantly less in the 6-month-old *Bdnf* CKO mice than controls (Figure 1D), suggesting that the reduction in sensory epithelial size is due to a loss of hair cells throughout the sensory epithelium.

Together these data show that sustained loss of *Bdnf* significantly reduces hair cell innervation and subsequently negatively impacts hair cell maintenance. However, many hair cells still survive despite the limited innervation and long-term loss of *Bdnf*, including the loss of all innervations of the canal cristae in many cases (Supplementary Figure 1).

### Loss of Vestibular Ganglion Neurons in *Bdnf* CKO Mice

**Figure 2:**
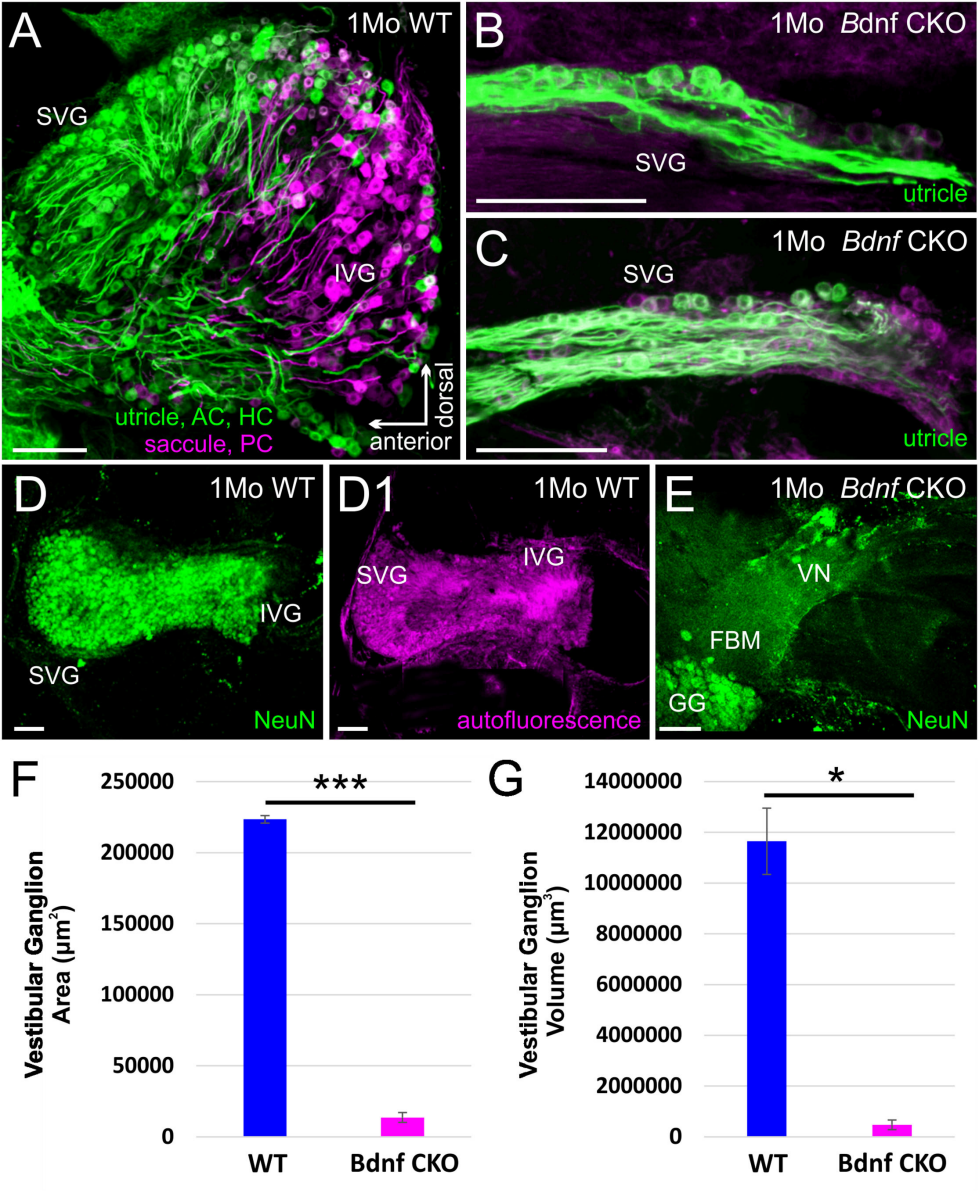
The vestibular ganglion is reduced following *Bdnf* loss. Lipophilic dyes placed into the utricle/anterior canal crista (AC)/horizontal canal crista (HC) (green) and the saccule/posterior canal crista (PC) (magenta) show a larger vestibular ganglion in control **(A)** than in *Bdnf* CKO 1-month-old mice **(B,C)**. The size difference is also visualized using NeuN antibody labeling **(D,E)**. This difference in size between the vestibular ganglion of control and *Bdnf* CKO mice is significant in the area **(F)** and the volume **(G)** of the ganglion. SVG, superior vestibular ganglion; IVG, inferior vestibular ganglion; VN, vestibular nerve; FBM, facial branchial motoneurons; GG, geniculate ganglion. Bars indicate 100 μm. *p < 0.05; ***p < 0.001.

The drastic reduction in sensory epithelial innervation was found to be reflected in the size of the vestibular ganglion in the *Bdnf* CKO mice (Figure 2), reflecting the work by others using *Bdnf* null mice (41–43, 51). By one month, the latest time point by which lipophilic dyes can be used to label neurons (Figures 2A–C) fluorescently, there was 93.9 ± 1.6% reduction in the vestibular ganglion area and a 95.9 ± 1.6% reduction in the vestibular ganglion volume in the *Bdnf* CKO mice when compared with controls (Figures 2F,G). The difference in size was confirmed with *NeuN* labeling (Figures 2D,E). Furthermore, applying dyes to the various vestibular sensory epithelia normally resulted in the labeling of vestibular ganglion neurons projecting to all sensory epithelia (Figure 2A). However, only a small population of vestibular ganglion neurons projecting to the utricle was labeled in the mutants (Figures 2B,C).

When lipophilic dyes were inserted into the brainstem and the cerebellum, we could not trace any fibers from the cerebellum or fibers from rhombomere 6 to the vestibular ganglia in the *Bdnf* CKO mice, unlike in controls (Supplementary Figure 2), suggesting neuronal fibers are lost or highly reduced.

### Reduced Vestibular Nucleus Innervation in *Bdnf* CKO Mice Has no Significant Effect on LVN Neurons or Cerebellar UBCs

**Figure 3:**
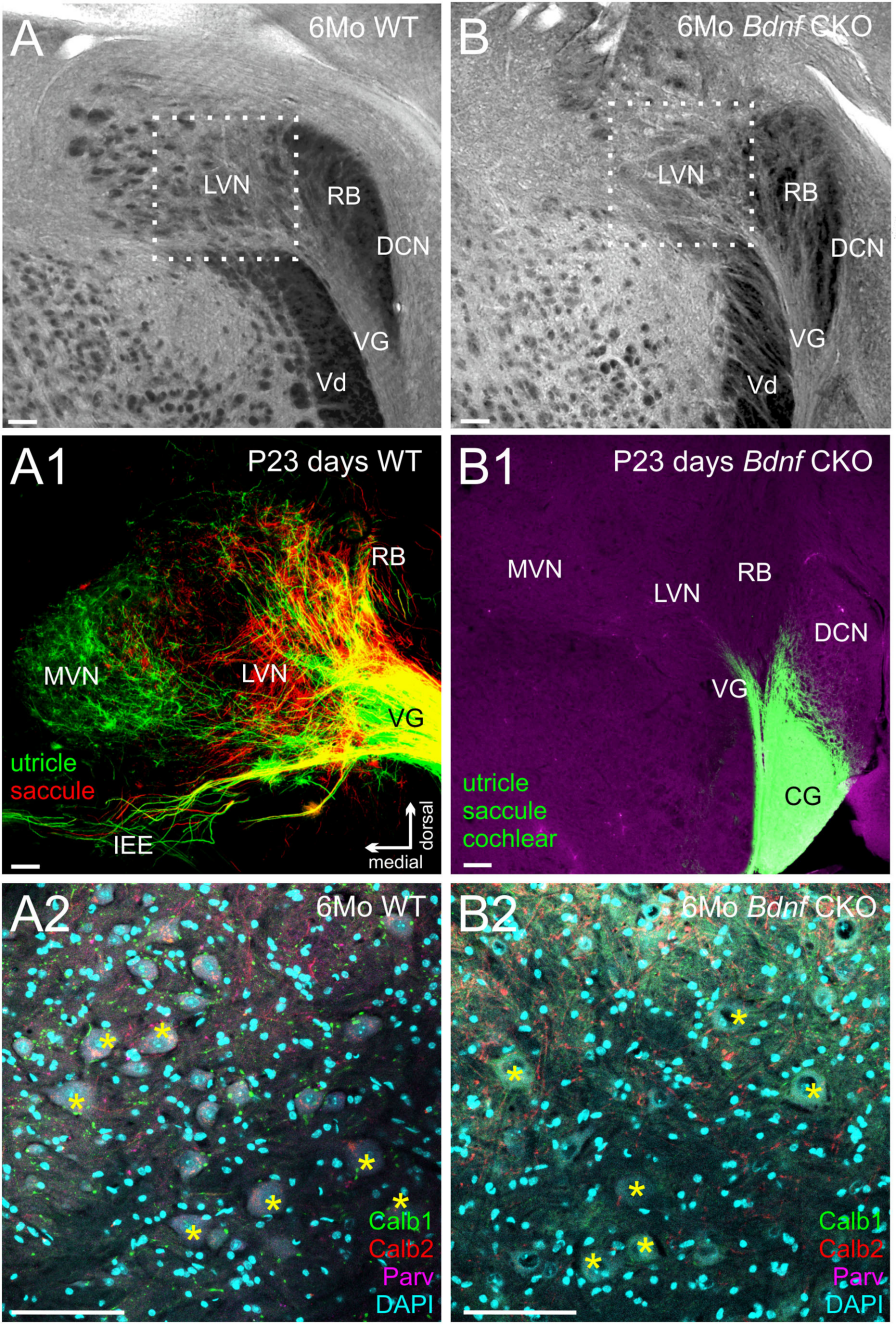
Reduction in the innervation of the LVN but not of LVN neurons following *Bdnf* loss. Labeling with tubulin in control **(A)** and *Bdnf* CKO **(B)** mice show a reduction of vestibular ganglion neuron innervation to the lateral vestibular nucleus (LVN) in the mutants, best visualized by the reduction in the gap between the restiform body (RB) and the descending trigeminal tract (Vd). This reduction is confirmed in P23 mice using lipophilic dyes [**(A1)**, Control; **(B1)**, *Bdnf* CKO]. Note that two different lipophilic dyes were implanted into the utricle [green; **(A1)**] and the saccule red, **(A1)** in controls compared with a single large dye implanted into the utricle, saccule, and cochlea in the *Bdnf* CKO **(B1)**. However, there was no significant difference in the number of large Deiters' neurons (yellow asterisks; labeled with Calb1, Calb2, and Parvalbumin) between the control **(A2)** and *Bdnf* CKO **(B2)** mice within the LVN (*p* > 0.05, *n* = 3, four animals, respectively). MVN, medial vestibular nucleus; DCN, dorsal cochlear nucleus; PVCN, posteroventral cochlear nucleus; VG, vestibular ganglion; SVG, superior vestibular ganglion, CG, cochlear ganglion; IEE, inner ear efferents; RB, restiform body; Vd, descending tract of the trigeminal fibers. Bars indicate 100 μm.

As a result of the reduced vestibular innervation and hair cell loss in *Bdnf* CKO mice, we consequently investigated the effect of *Bdnf* loss in vestibular target regions of the brain. Within the brain, vestibular ganglion neurons pass through the gap between the restiform body and the trigeminal tract and terminate in the LVN. In control mice this gap is rather large (Figure 3A) compared with that of *Bdnf* CKO mice (Figure 3B), suggesting diminished central innervation and is consistent with the reduced numbers of vestibular ganglion neurons. At the latest time point lipophilic dyes reliably label neuronal tracts (P23), the reduction in central vestibular projection for *Bdnf* CKO mice was confirmed (Figure 3A1,B1). Few vestibular ganglion neuron fibers were observed entering the LVN in rhombomere 4 in the *Bdnf* CKO mice (Figure 3B1), whereas in controls, the vestibular ganglion neurons can be traced to reach the LVN and MVN from the utricle and saccule (Figure 3A1).

To determine whether this loss of *Bdnf* and reduced innervation negatively affected the large Deiters' neurons of the LVN, we quantified the number of these cells in serial coronal brain sections. Using antibodies against Calb1, Calb2, and Parvalbumin, as well as DAPI labeling of nuclei (Figure 3A2–3B2), we found that the mean number of large Deiters' neurons per LVN section in the *Bdnf* CKO mice (11.3 ± 2.3) compared with that in control mice (13.6 ± 1.2) were not statistically different (*p* > 0.05, *n* = 4, 3, respectively). These results suggest that a loss of *Bdnf* and reduced vestibular ganglion neuron innervation did not affect Deiters' neuron survival in 6-month-old mice. The limited remaining vestibular ganglion neuron innervation and/or input from other neurons was sufficient to prevent the loss of these large LVN neurons for at least 6 months.

Knowing that vestibular ganglion fibers could not be traced to the cerebellum, we next examined the survival of the UBCs in the nodulus and uvula. Using Calb1 to label Purkinje cells and Calb2 to label UBCs, we quantified the numbers of each within 2,500 μm^2^ representative areas of a single optical parasagittal section of the nodulus in both controls and *Bdnf* CKO mice. As the results in the LVN, there was no significant difference in the mean numbers of UBCs between control (4.5 ± 0.5) and *Bdnf* CKO mice (3.5 ± 0.3) within the representative areas (*p* > 0.05, *n* = 4,4, respectively). Similarly, there was no significant difference in the number of Purkinje cells between control (15.8 ± 2.1) and *Bdnf* CKO mice (16.1 ± 1.2) along a 400 μm length of the nodule (*p* > 0.05, *n* = 4,4, respectively). Together these results suggest that the survival of central target cells in *Bdnf* CKO mice may be a consequence of their interconnectivity with other CNS neurons.

## Discussion

**Figure 4:**
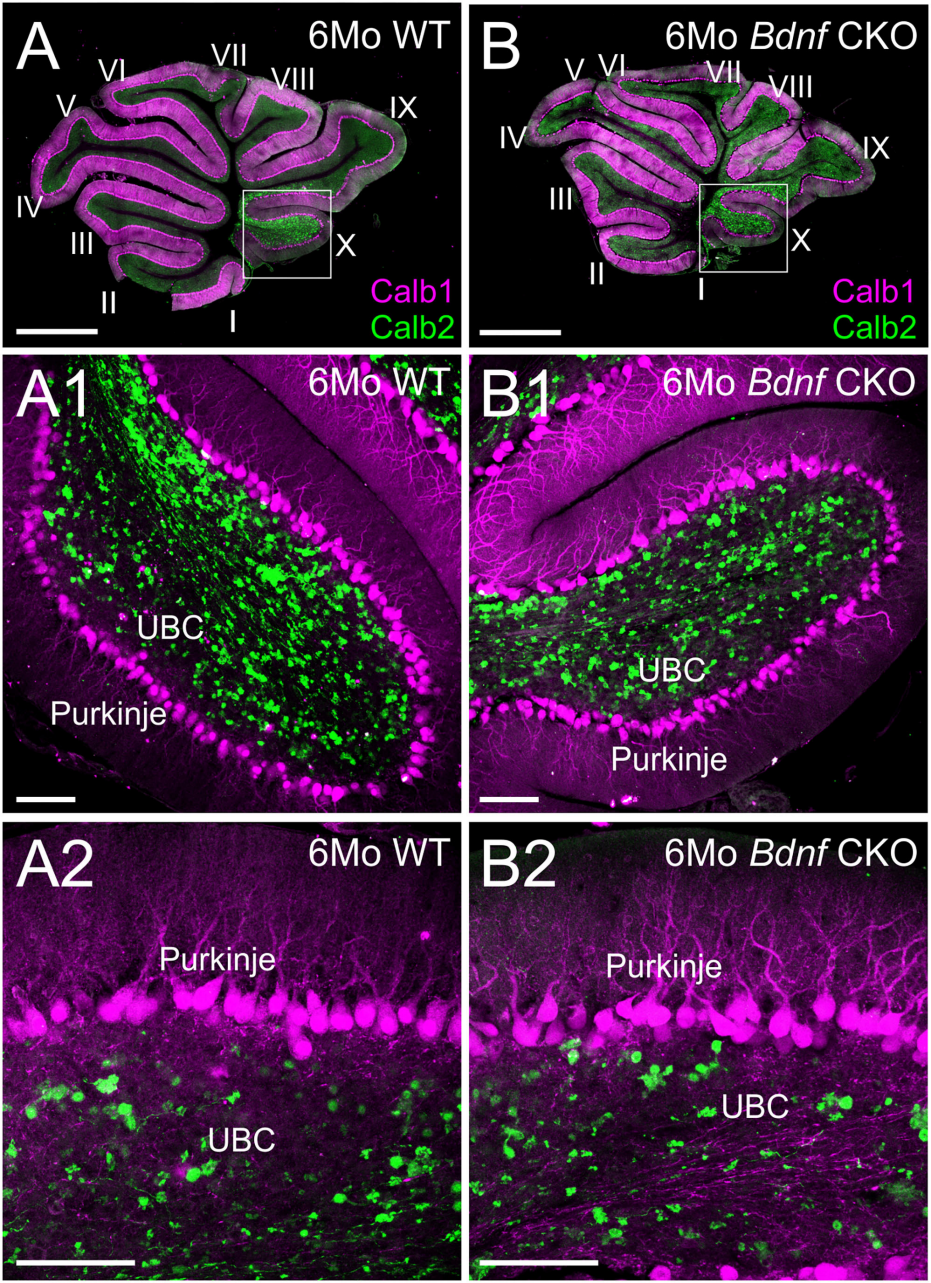
Purkinje cells and unipolar brush cells are not affected following *Bdnf* loss. Overviews of parasagittal cerebellar sections from 6-month-old control **(A)** and *Bdnf* CKO **(B)** mice labeled with Calb1 (magenta) and Calb2 (green). Higher magnifications of control **(A1,2)** and *Bdnf* CKO **(B1,2)** mice show no difference in unipolar brush cells (UBC) and Purkinje cells between controls and mutants (*p* > 0.05, *n* = 4, 4, respectively). Bars indicate 100 μm.

Our data expand upon previous studies investigating the effects of the loss of *Bdnf* (41, 44). To circumnavigate that *Bdnf* null mice die only a few weeks after birth (41), we used *Pax2*-cre to knock out *Bdnf* conditionally. The strategy allowed us to study the longer-term effects of *Bdnf* loss on the vestibular system. Using our mice with *Bdnf* conditionally knocked out, we found that a reduction of vestibular ganglion neurons is accompanied by a decrease in the number of vestibular hair cells and a reduction of the size of vestibular end organs by 6 months (Figures 1, 2). In addition, we found that central processes of the few remaining vestibular ganglion neurons can be traced to the LVN but cannot be traced beyond that (Figure 3) unlike control vestibular ganglion neurons that can be treaced to the LVN and MVN (Figure 3). Moreover, we showed that in the near absence of LVN input by vestibular ganglion neurons, there is no effect on the large Deiters' neuron survival in the LVN, nor is there an effect on the UBCs in the nodulus and uvula of the cerebellum (Figures 3, 4).

The severe loss of vestibular ganglion neurons in the *Bdnf* CKO mice (Figures 1, 2) was comparable to that observed in *Bdnf* null mice (41–43, 51). Our results validate that the CKO model sufficiently recapitulates the *Bdnf* null phenotype for vestibular ganglion neurons. The loss of innervation to the peripheral vestibular end organs was not uniform. Vestibular ganglion cells that project to the cerebellum innervate the middle section of the saccule and the lateral utricle (16, 21, 22). These regions preferentially lose innervation in the *Bdnf* CKO mice (Figure 1). Consistent with this finding was our inability to trace vestibular projections from dyes implanted into the cerebellum. While a few fibers remained to innervate parts of the vestibular end organs in the *Bdnf* CKO mice at 6 months, this innervation was insufficient to prevent vestibular hair cell loss (Figure 1). Our findings were similar to the loss of vestibular hair cells following the near complete loss of innervation in mice in which both alleles of *Bdnf* and one allele of *Ntf3* were conditionally knocked out (38). However, the loss of neurons and hair cells is not as profound when only *Bdnf* is conditionally knocked out. The conditional loss of both alleles of *Bdnf* and *Ntf3* or both receptors resulting in no innervation and no surviving hair cells (38) suggests a minor role for *Ntf3* in the vestibular system. Thus the two pairs of neurotrophins and neurotrophin receptors are essential and sufficient for vestibular neuron viability (51, 52). Whether specific vestibular ganglion neurons are more susceptible to *Bdnf* loss alone than other neurons remains to be determined. Extirpation of the labyrinth (53) halts the spontaneous activity of LVN neurons (54). However, over time, this unilateral vestibular loss is partially compensated for in humans (55) and mice (56). This compensation of vestibular function requires *Bdnf* signaling (57). In the absence of *Bdnf* , null mice cannot compensate due to the bilateral loss of most vestibular ganglion neurons and cannot be easily tested behaviorally with typical rotarods and gait analysis systems like the Noldus CatWalk (58, 59) as they fall (rotarod) or stay put (Catwalk). Likewise, we know that an incomplete loss of vestibular ganglion neurons following *Neurod1* conditional knockout shows a defect in behavior (60). To fully describe vestibular compensation after the loss of *Bdnf* would require measuring vestibular sensory-evoked potentials (VsEPs) (58).

Unlike the loss of hair cells at the periphery, there was no loss of central target neurons in either the LVN (Figure 3) or the cerebellum (Figure 4) following vestibular ganglion neuronal loss in *Bdnf* CKO mice at 6 months of age. The most plausible explanation is that the interconnectivity of these target neurons with other central neurons could compensate for the loss of vestibular ganglion neurons (16, 18, 21, 27–32). During normal aging, LVN neurons are lost (1, 33) and *Bdnf* expression declines (61). It would be important to determine whether a loss of *Bdnf* results in a premature loss of central neurons. This would require reliably raising *Bdnf* CKO mice to at least 25 months of age, the time point at which the loss of large Deiters' neurons in the LVN begins to occur in wild-type mice (33). While conducting this study, we had two animals survive until ~1.5 years of age that had both alleles of *Bdnf* conditionally deleted but had, in addition, one allele of *Ntf3* lost (Pax2-cre; *Bdnf*^*f* /*f*^*, Ntf3*^*f* /+^, Supplementary Figures 1, 3). At this time point, there were fewer large Deiters' neurons observed in the LVN of these mice (Supplementary Figure 3). Nevertheless, the extent that the additional loss of one allele of *Ntf3* adds to the *Bdnf* phenotype and subsequent loss of LVN neurons at 1.5 years is unknown. However, this may indicate that loss of *Bdnf* could eventually lead to an earlier decline of the Deiters' neurons in the LVN than observed during normal aging. Furthermore, there was a further reduction in peripheral innervation (Supplementary Figure 1C), but again whether this loss is due to aging or the loss of an allele of *Ntf3* is unknown. However, there was no perceived effect on the cerebellum in these mice (Supplementary Figure 3).

**Figure 5:**
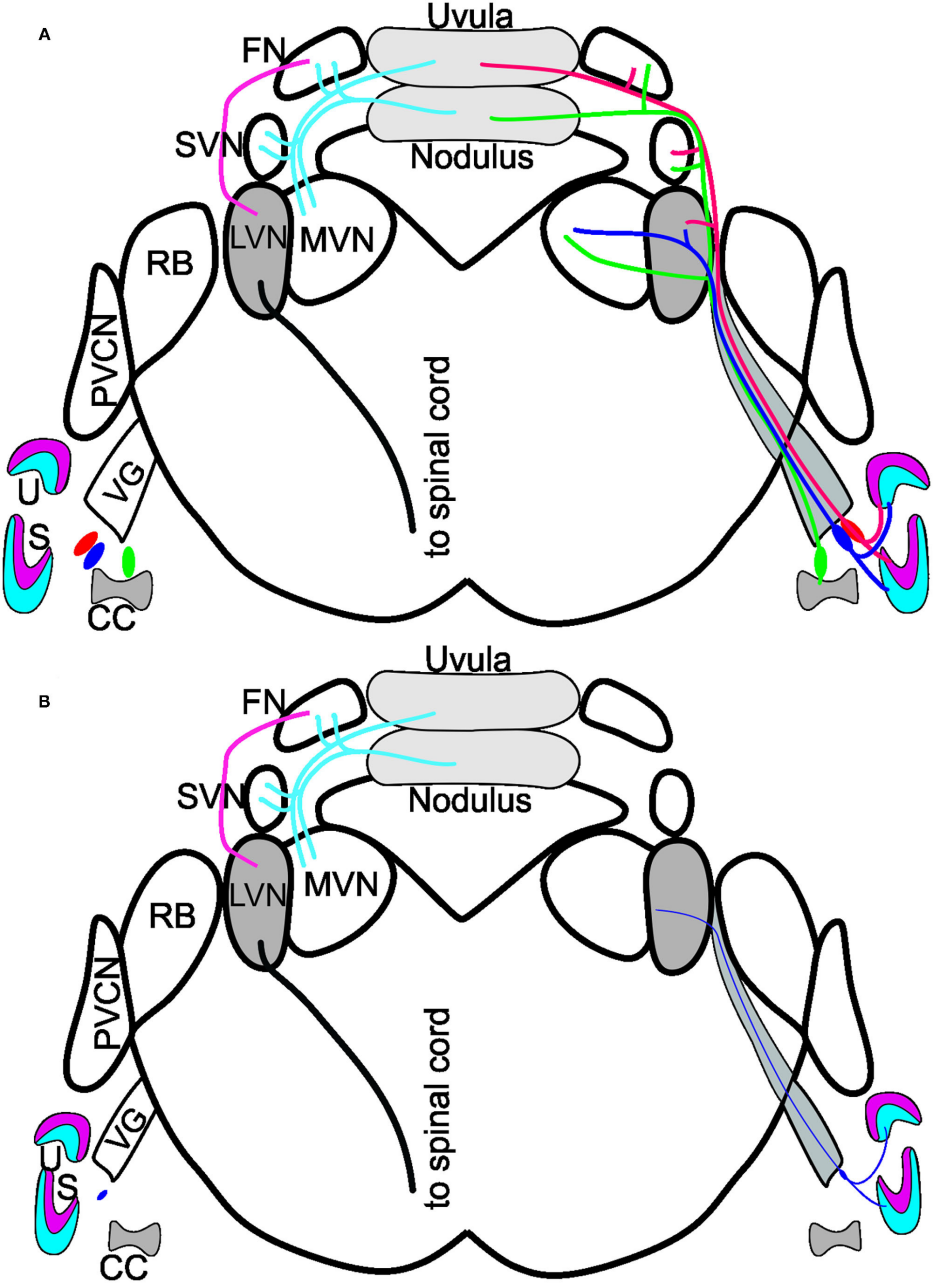
Summary of *Bdnf* CKO effects on the vestibular system. **(A)** Vestibular ganglion neurons innervating the utricle (U; red/blue) and saccule (S; red/blue) and canal crista (CC; green) of control mice project to the various vestibular nuclei: lateral vestibular nucleus (LVN), medial vestibular nucleus (MVN) and the superior vestibular nucleus (SVN). The fibers continue to have collateral branches with the fastigial nucleus (FN) before the fibers end in the uvula (red) and the nodulus (green) of the cerebellum. Dyes injected into the cerebellum reach the FN, SVN, and MVN but do not expand to innervate the LVN. In contrast, a strong projection from the FN to the LVN exists, giving rise to a spinal cord output. Note that the cerebellum and fastigial injections are bilateral. **(B)** In the *Bdnf* CKO mice, vestibular ganglion neurons are reduced to the various end-organs (thin purple fiber). Injection of dyes into the vestibular ganglion neurons at the periphery showed only a few fibers projecting to the LVN. Though, at 6-months of age, there was no reduction in LVN neurons or neurons in the nodulus. Modified after (16, 18, 27, 29).

In summary, while the loss of vestibular ganglion neurons in *Bdnf* CKO mice leads to a significant loss of hair cells at the periphery, there is no effect on central targets in the LVN or cerebellum at 6 months (Figure 5). It suggests that the latter target cells likely receive compensatory input from other neurons. Whether the central target cells would ultimately die prematurely in the absence of *Bdnf* remains to be determined.

## Data Availability Statement

The original contributions presented in the study are included in the article/Supplementary Material, further inquiries can be directed to the corresponding author.

## Ethics Statement

The animal study was reviewed and approved by IACUC #0021971.

## Author Contributions

All authors listed have made a substantial, direct, and intellectual contribution to the work and approved it for publication.

## Funding

We thank members of our laboratories for their comments on this manuscript. Grants to ENY and BF supported this work from the National Institutes of Health (DC016099, DC015252, DC015135, AG060504, AG051443).

## Conflict of Interest

The authors declare that the research was conducted in the absence of any commercial or financial relationships that could be construed as a potential conflict of interest.

## Publisher's Note

All claims expressed in this article are solely those of the authors and do not necessarily represent those of their affiliated organizations, or those of the publisher, the editors and the reviewers. Any product that may be evaluated in this article, or claim that may be made by its manufacturer, is not guaranteed or endorsed by the publisher.

## Supplementary Material

Supplementary Figure 1 Peripheral Innervation. **(A,B2)** The various canal cristae of 6-month-old *Bdnf* CKO mice: anterior canal cristae [AC; **(A, A1, B)**]; posterior canal cristae [PC; **(A2,3, B2)**]; horizontal canal cristae [HC; **(B1)**]. **(C)** Saccule of a 1.5-year-old *Bdnf* CKO mouse showing reduced innervation and an absence of innervation from the superior vestibular ganglion. IVG, inferior vestibular ganglion. Bars indicate 100 μm.

Supplementary Figure 2 Vestibular ganglion neurons project to either the brainstem (red) or cerebellum (green), as shown with the injection of lipophilic dyes into the target regions in control mice (shown). Injection of dyes into rhombomere 5 of the brainstem or the cerebellum of *Bdnf* CKO mice did not label any vestibular ganglion neurons. SVG, superior vestibular ganglion; IVG, inferior vestibular ganglion. Bar indicates 100 μm.

Supplementary Figure 3 Tubulin labeling of control **(A)** and *Pax2*-cre; *Bdnf*^*f*/*f*^ *Ntf3*^*f*/+^ **(B)** 1.5-year-old mice show a reduction of vestibular ganglion (VG) innervation to the lateral vestibular nucleus (LVN). Tubulin (green), neurofilament (magenta), and DAPI (cyan or magenta) labeling within the LVN show that, compared with control **(A1)**, there is a reduction of large Deiters' neurons [LVN in **(A1)** and **(B1)**] in the *Pax2*-cre; *Bdnf*^*f*/*f*^ *Ntf3*^*f*/+^ mice **(B1)**; however, conclusions on the timeline of LVN neuron loss cannot be fully drawn due to the absence of an allele of *Ntf3* in addition. We compare the cerebellum in control and *Bdnf* CKO *Ntf3* Het mice that show no reduction in either the nodulus or the uvula **(A2,B2)**. LVN, lateral vestibular nucleus; MVN, medial vestibular nucleus; DCN, dorsal cochlear nucleus; PVCN, posteroventral cochlear nucleus; RB, restiform body; Vd, descending tract of the trigeminal. Bars indicate 100 μm in **(A,A2,B,B2)** and 10 μm in **(A1,B1)**.
