# Supplementary figures and images for: Sustained Loss of Bdnf Affects Peripheral but Not Central Vestibular Targets
Source: Front Neurol. 2021 Dec 16;12:768456. doi: 10.3389/fneur.2021.768456 (PMC8716794; doi:10.3389/fneur.2021.768456)

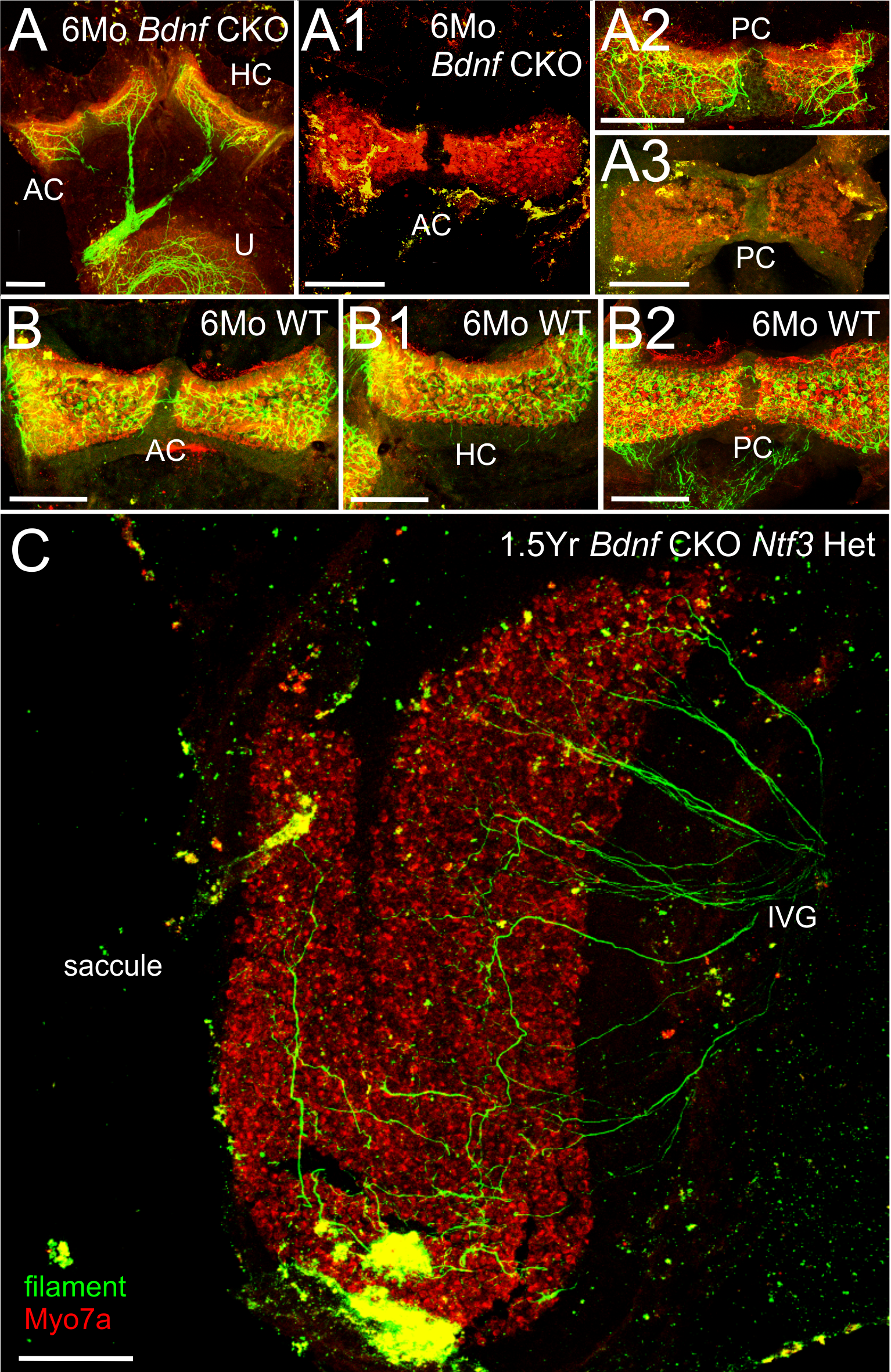

Supplement: Supplementary Figure 1 — Peripheral Innervation. (A,B2) The various canal cristae of 6-month-old Bdnf CKO mice: anterior canal cristae [AC; (A, A1, B)]; posterior canal cristae [PC; (A2,3, B2)]; horizontal canal cristae [HC; (B1)]. (C) Saccule of a 1.5-year-old Bdnf CKO mouse showing reduced innervation and an absence of innervation from the superior vestibular ganglion. IVG, inferior vestibular ganglion. Bars indicate 100 μm. [file Image_1.TIF]

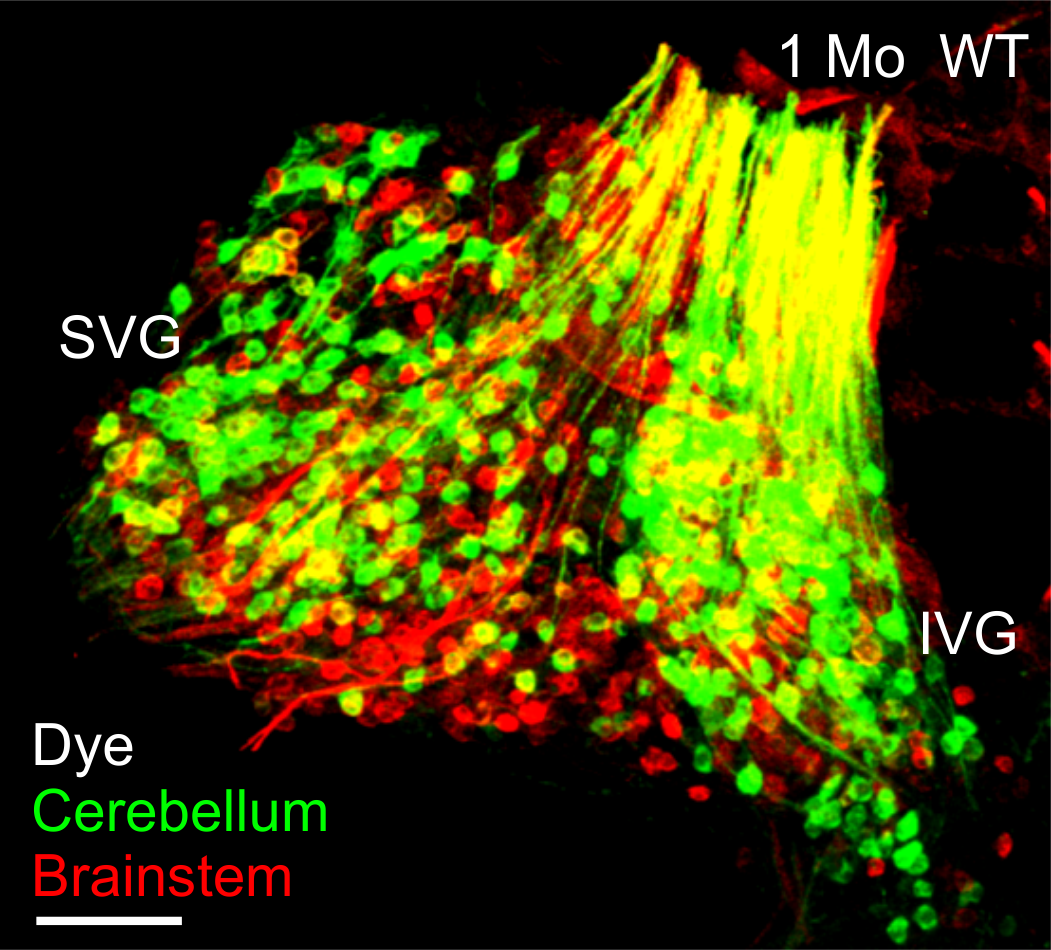

Supplement: Supplementary Figure 2 — Vestibular ganglion neurons project to either the brainstem (red) or cerebellum (green), as shown with the injection of lipophilic dyes into the target regions in control mice (shown). Injection of dyes into rhombomere 5 of the brainstem or the cerebellum of Bdnf CKO mice did not label any vestibular ganglion neurons. SVG, superior vestibular ganglion; IVG, inferior vestibular ganglion. Bar indicates 100 μm. [file Image_2.TIF]

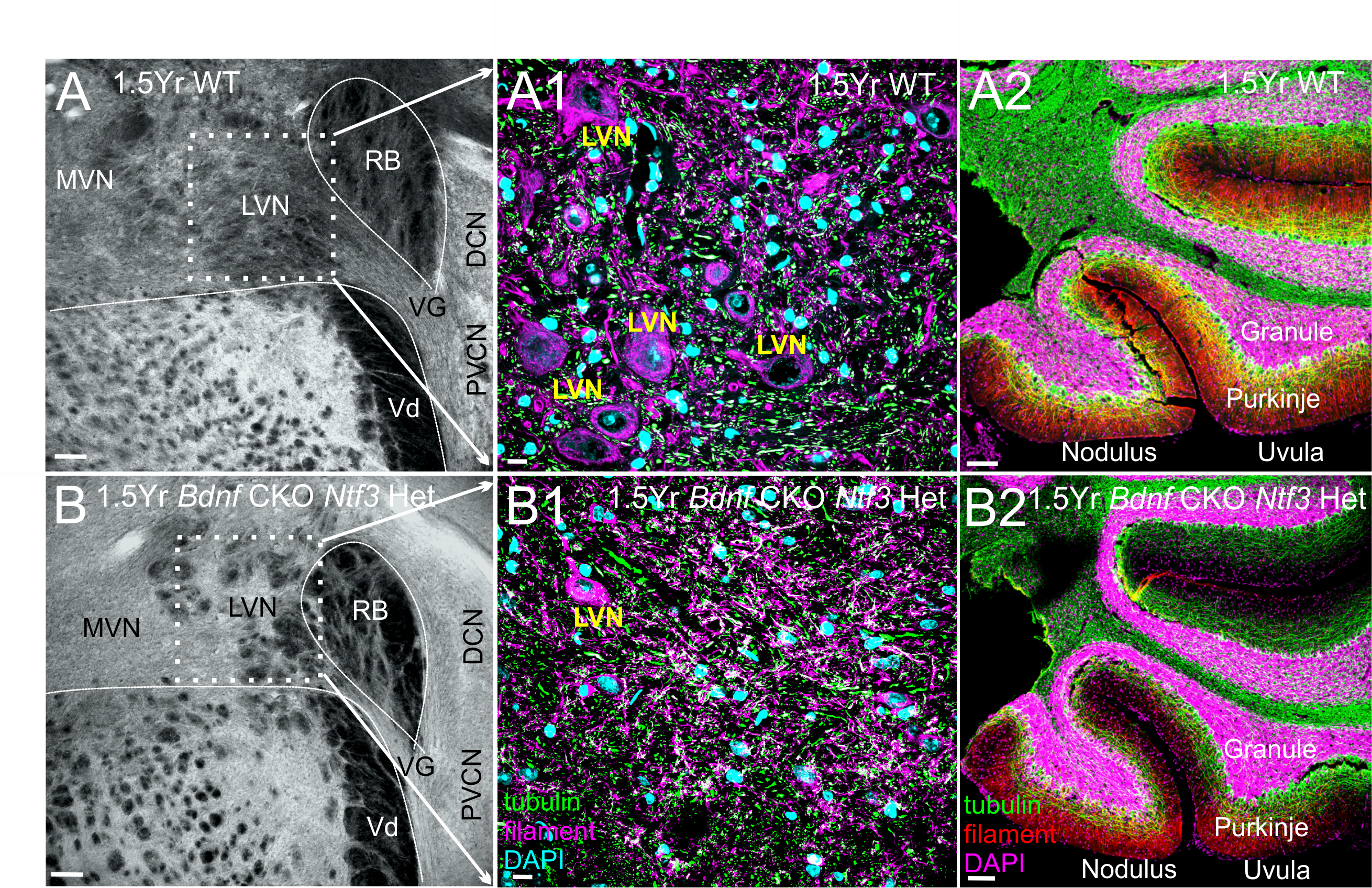

Supplement: Supplementary Figure 3 — Tubulin labeling of control (A) and Pax2-cre; Bdnff/f Ntf3f/+ (B) 1.5-year-old mice show a reduction of vestibular ganglion (VG) innervation to the lateral vestibular nucleus (LVN). Tubulin (green), neurofilament (magenta), and DAPI (cyan or magenta) labeling within the LVN show that, compared with control (A1), there is a reduction of large Deiters' neurons [LVN in (A1) and (B1)] in the Pax2-cre; Bdnff/f Ntf3f/+ mice (B1); however, conclusions on the timeline of LVN neuron loss cannot be fully drawn due to the absence of an allele of Ntf3 in addition. We compare the cerebellum in control and Bdnf CKO Ntf3 Het mice that show no reduction in either the nodulus or the uvula (A2,B2). LVN, lateral vestibular nucleus; MVN, medial vestibular nucleus; DCN, dorsal cochlear nucleus; PVCN, posteroventral cochlear nucleus; RB, restiform body; Vd, descending tract of the trigeminal. Bars indicate 100 μm in (A,A2,B,B2) and 10 μm in (A1,B1). [file Image_3.TIF]
